# Urinary Concentrations of Toxic and Essential Trace Elements among Rural Residents in Hainan Island, China

**DOI:** 10.3390/ijerph111213047

**Published:** 2014-12-12

**Authors:** Yosuke Inoue, Masahiro Umezaki, Hongwei Jiang, Dandan Li, Jianwei Du, Yuming Jin, Bin Yang, Bai Li, Yufeng Li, Chiho Watanabe

**Affiliations:** 1 Department of Human Ecology, Graduate School of Medicine, the University of Tokyo, 7-3-1 Hongo, Bunkyo-ku, Tokyo 113-0033, Japan; E-Mails: umezaki@humeco.m.u-tokyo.ac.jp (M.U.); chiho@humeco.m.u-tokyo.ac.jp (C.W.); 2 Research Institute for Humanity and Nature, 457-4 Motoyama, Kamigamo, Kita-ku, Kyoto 603-8047, Japan; E-Mail: jianghw@gmail.com; 3 Hainan Provincial Center for Disease Control and Prevention, 44 Haifu Road, Haikou, Hainan 57023, China; E-Mails: adanleee0898@gmail.com (D.L.); djwei22@sohu.com (J.D.); jym1030@126.com (Y.J.); ybdoctor@163.com (B.Y.); 4 Institute of High Energy Physics, Chinese Academy of Science, 19B Yuquan Road, Shijingshan District, Beijing 100049, China; E-Mails: libai@ihep.ac.cn (B.L.); liyf@ihep.ac.cn (Y.L.)

**Keywords:** toxic elements, heavy, trace elements, urine, economic development, China

## Abstract

*Background*: Toxic element exposure and essential trace element consumption may have changed after the Chinese economy transformed to a market-oriented system. The objectives of this study were to measure urinary concentrations of toxic (arsenic, cadmium, lead) and essential trace (selenium, zinc, copper) elements among rural residents in Hainan, China and to examine if variations in economic development are linked to differences in toxic and trace element exposure. *Methods*: We conducted a questionnaire-based survey and undertook anthropometric measurements of residents aged ≥20 years (*n* = 599). Urinary samples were collected and analyzed using inductively coupled plasma mass spectrometry. *Results*: The median (μg/g creatinine) element concentrations were: arsenic, 73.2; cadmium, 1.8; lead, 3.1; selenium, 36.5; zinc, 371; and copper, 11.0. Intra-community variation in element concentrations was explained by age (arsenic, cadmium, zinc and copper), sex (arsenic, cadmium and selenium: higher in females; zinc: higher in males), body mass index (cadmium) and individual involvement in the market economy as indexed by agrochemical use (lead and selenium). The degree of community-level economic development, which was determined by the proportion of people living in better housing among the study communities, was positively associated with cadmium concentration. *Conclusions*: The degree of community-level economic development was positively associated with urinary cadmium concentration while individual involvement in the market economy was positively associated with lead and selenium.

## 1. Introduction

Exposure to toxic elements such as arsenic (As), cadmium (Cd), and lead (Pb) is associated with various health problems. The International Agency for Research on Cancer (IARC) has categorized As and Cd as group 1 (carcinogenic to humans) [1] and Pb as group 2A (probably carcinogenic to humans) toxins [2]. Exposure to As is also associated with cardiovascular and neurological disorders [3], and exposure to Cd can result in kidney damage [4]. Impaired neurological development has been linked to Pb exposure [5,6].

Human populations have been exposed to these toxic elements through anthropogenic sources, especially since the time of the Industrial Revolution. An estimated 40% of the global atmospheric input of As results from anthropogenic activities, such as copper smelting and coal combustion [7]. Cd has been extracted for non-ferrous metal mining and the manufacture of Cd-containing products since the beginning of the 20th century, resulting in Cd contamination of local environments [8]. The amount of Pb circulating in local, regional, and global ecosystems has also increased due to human activities, such as the use of leaded gasoline and biomass combustion [9].

Selenium (Se), zinc (Zn), and copper (Cu) are essential for sustaining human life as catalytic or structural components of larger molecules [10]. Se has antioxidant and anti-inflammatory effects, and influences the production of active thyroid hormone [11]. Cu acts as a cofactor for oxidative metalloenzymes, including lysyl oxidase, cytochrome c oxidase, and superoxide dismutase [12]. More than 100 enzymes involved in energy metabolism and in transcription and translation are Zn dependent [13].

Dietary Se, Zn, and Cu deficiencies are related to health disorders [14]. Se deficiency results in suboptimal selenoenzyme activity [15], and low dietary Se levels have been associated with Keshan disease, an endemic cardiomyopathy in northeastern China [11]. Zn deficiency has been reported to affect the immune system, taste, and smell and to impair DNA synthesis, and Cu deficiency results in anemia, leukopenia, and skeletal demineralization [16]. Because the consumption of meat, which usually contains Se, Zn, and Cu, doubled worldwide between 1950 and 2009 [17], the concentrations of these elements now being consumed are expected to increase, even among people in developing countries.

As a part of a cross-border research project (Environment Research in Rural Asia—ENVRERA), which aimed to elucidate the health consequences of rapid lifestyle change, especially as regards the introduction of chemical substances among rural residents in seven countries in the Asia-Pacific region, this study investigated the urinary excretion of toxic (As, Cd, and Pb) and essential trace (Se, Zn, and Cu) elements in several rural farming communities of Hainan Island, China, where economic development has been mainly driven by the introduction of modern agriculture and partly, by the development of tourism. 

Rapid transformation to a market economy since the 1980s has been accompanied by significant lifestyle changes in rural China [18,19,20]. In the case of Hainan Island, artificially produced chemicals (*i.e.*, pesticides, herbicides, fungicides, fertilizers, and medicines) and industrial goods (*i.e.*, gasoline, batteries, paints, and building materials) have been introduced into the rural communities [21]. This population has begun to consume more meat, fish, and processed foods. This study defined those who have been affected by these lifestyle changes to a greater degree as being in a more economically developed position and explored whether such transitions in chemical exposure and diet have been accompanied by changes in exposure levels to toxic elements or the consumption of essential trace elements.

The urinary concentrations of As, Cd, and Pb are widely used biomarkers of exposure. Urinary As and Se concentrations are known to reflect recent exposure/intake levels, the durations of which have been suggested to be up to 1 week for As [22,23] and 24 h for Se [24]. The urinary Cd concentration is correlated with the amount of Cd accumulated in the kidneys, and thus probably reflects the cumulative dose of Cd exposure [25].

The urinary concentrations of Pb, Zn, and Cu also change in response to different exposure/intake levels, although they are less frequently used as biomarkers. The urinary Pb concentration is a good alternative to the blood Pb level for environmental health research in highly polluted areas [26]. The urinary Zn excretion level has been observed to increase after Zn supplementation trials [27]. Cu supplementation (7.8 mg/day) resulted in a significant increase in the urinary excretion level [28].

This study aimed to (1) describe the urinary concentrations of toxic (As, Cd, and Pb) and essential trace (Se, Zn, and Cu) elements among rural residents of Hainan Island, China; and (2) to examine if variations in economic development are linked to differences in toxic and trace element exposure among them.

## 2. Material and Methods

### 2.1. Study Communities

Hainan Island is located to the south of mainland China. The island has a subtropical climate with an average temperature of 22.8–25.8 °C and annual precipitation of 900–2500 mm [29]. The population of the province was 8.6 million in 2010. Nearly all of the residents belong to either the Han (83%) or Li (16%) ethic groups.

Hainan Island, formerly part of Guangdong Province, became an independent province and was designated as a special economic zone in 1988. Since then, it has received large investments from mainland China and overseas, especially to the tourism and agriculture sectors. Its subtropical climate is suitable for the cultivation of cash crops such as coffee, banana, litchi, mango, and longyan, and the island is ideal for tourism due to its extensive sandy beaches. Economic development projects initially focused on the coastal areas and were then gradually extended to inland rural communities by the end of the 1990s.

**Figure 1:**
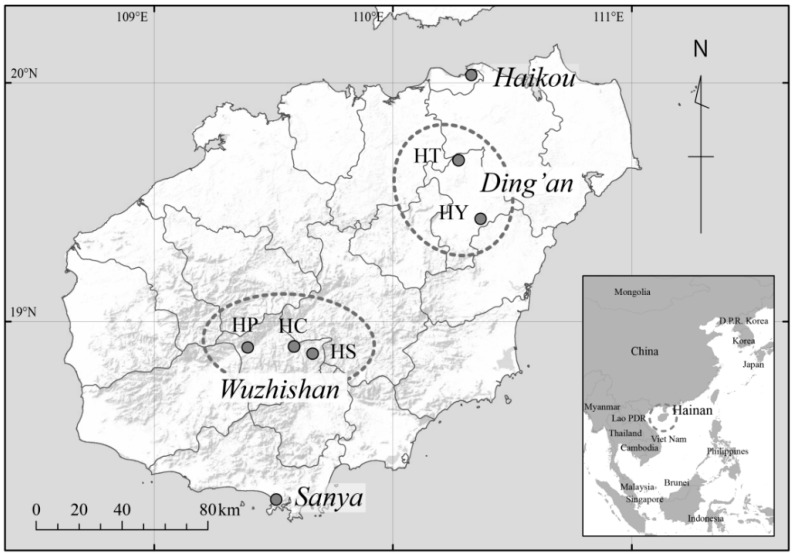
Map of Hainan Island.

Figure 1 shows the location of the regions where the present study was undertaken. Two communities were located in Ding’an County (Communities HT and HY; Han communities) and three in Wuzhishan City (Communities HC, HP and HS; Li communities). Our assessment of the degree of community-level economic development was based on the proportion of those who live in better housing. Specifically, the extent of economic development was determined by calculating the proportion of households living in traditional housing compared to those living in concrete housing, which gave us the following sequence: HC (least economically developed), HP, HY, HT, and HS (most economically developed) (Table 1). This ordering was also in accordance with the types of toilet possession in these communities. We assumed that these communities had similar levels of economic development in the 1980s, but became economically stratified during the developmental process that has been occurring since the 1990s [30]. In the course of economic development, variation emerged in the extent to which individual residents successfully established market economies within their communities [21,31,32]. For example, people with successful cash crop–based agricultural endeavors had more opportunities to use agrochemicals and agricultural machinery to improve production, and they also consumed more meat, fish, and processed foods [21].

**Table 1:** Household characteristics by community ^a^.

Characteristics	HC (*n* = 64)		HP (*n* = 38)		HY (*n* = 91)		HT (*n* = 104)		HS (*n* = 40)	
	*n*	[%]	*n*	[%]	*n*	[%]	*n*	[%]	*n*	[%]
Housing Type ^b^										
Concrete	0	[0]	6	[15.8]	9	[9.9]	30	[29.1]	15	[37.5]
Chinese-Tiled	0	[0]	1	[2.6]	82	[90.1]	72	[69.9]	22	[55.0]
Traditional	59	[100]	31	[81.6]	0	[0.0]	1	[1.0]	3	[7.5]
Toilet ^c^										
Inside	1	[1.6]	3	[8.1]	62	[68.9]	77	[74.8]	35	[92.1]
Outside	61	[98.4]	34	[91.9]	28	[31.1]	26	[25.2]	3	[7.9]

^a^ HC, HP, HY, HT and HS are names of the study communities; ^b^ Housing was classified into: concrete housing, plastered housing with Han-style roof tiles, and traditional housing (wooden or bamboo housing); ^c^ The toilet inside category includes toilets constructed for that purpose. The toilet outside category includes defecation or urination in any of the following places: river, stream, creek, sea, yard, bush, and forest.

### 2.2. Field Survey

A field survey was conducted in October 2008. The authors employed a convenience sampling method, in which people present in the targeted villages were invited to participate during the survey period. We collected urine samples since this collection method is not invasive and was deemed acceptable by respondents in the (widely differing) study communities in the seven counties in the ENVRERA project. Urine samples were collected in the morning and immediately stored at −20 °C. Together with local trained staff, we conducted a questionnaire-based survey and undertook the anthropometric measurement of residents aged ≥20 years (*n* = 599). Height and weight were measured using a standard anthropometer (minimum unit, 0.1 cm; DKSH Switzerland Ltd., Zurich, Switzerland) and a digital scale (minimum unit, 0.1 kg; HD-654; Tanita Corp., Tokyo, Japan), respectively. Body mass index (BMI) was calculated by dividing an individual’s weight (kg) by the square of their height (m^2^). Participants were asked questions regarding their demographic attributes (age and sex) and agrochemical use in the past year. Specifically, they responded to the questions “Did you use agrochemicals, herbicides or pesticides in the past year?”. All interviews were conducted in Mandarin Chinese or the Hainan dialect.

### 2.3. Measurement of Toxic and Trace Elements

Frozen urine was defrosted in a laboratory of the Chinese Academy of Science, Beijing, China, after being stored at −80 °C at the Hainan Provincial Center for Disease Control and Prevention (CDC). Following homogenization, aliquots of urine (1 mL) were diluted by one-tenth with a nitric acid solution (2% v/v) containing internal standards (scandium, germanium, yttrium, and terbium) at a final concentration of 25 μg/L. Element concentrations in urine samples were determined using an inductively coupled plasma mass spectrometer (Thermo X; Thermo Fisher Scientific Inc., Franklin, MA, USA). The mass of interest (m/z) for each analysis was: As = 75, Cd = 111, Pb = 208, Zn = 68, Se = 80, and Cu = 65. Seronorm Trace Elements Urine (Seronorm Pharmaca, Billingstad, Norway) and SPEX QC-21 (SPEX CertiPrep, Metuchen, NJ, USA) were used as a reference material and standard solution, respectively. The recoveries for As, Cd, Pb, Se, Zn and Cu were between 90%–110%. Observed values fell within the range of certified values.

Limits of quantification (LOQ) were calculated as ten times as large as standard deviations (SD) of the blank measures: 0.430 µg/L for As, 0.066 µg/L for Cd, 0.076 µg/L for Pb, 0.320µg/L for Se, 0.165 µg/L for Zn, and 0.066 µg/L for Cu. There was one sample which was below one of these LOQs, which was substituted with half the value of the LOQ. The repeatability in terms of relative standard deviation (R.S.D. %, *n* = 10) was 6%. Urinary creatinine levels were measured at Hainan Provincial People’s Hospital using a chemistry analyzer (Cobas 6000; Roche, Basel, Switzerland). 

### 2.4. Statistical Analysis

A multi-level regression model was used to examine the association of urinary element concentrations with individual variables (*i.e.*, age, sex, BMI, educational attainment [years of schooling: <9 years, 9 years (equivalent to graduation from middle school), and >9 years], and individual involvement in the market economy) and community-level variables (*i.e.*, community levels of economic development and community ethnicity [Han or Li]. The degree of individual involvement in the market economy was measured by agrochemical use in the previous year (yes or no). We assumed that only participants involved in cash-crop agriculture would spray costly agrochemicals; agrochemical usage in the previous year thus shows the extent of individuals’ involvement in cash-crop agriculture (the major activity for earning cash). Because the prevalence of smoking was far lower in females (0.2%) than in males (68%), associations between smoking and urinary element concentrations were investigated only in male participants. 

Urinary element concentrations were adjusted using creatinine concentrations and log transformed before the analyses. Eight participants with missing information for one or more variables were omitted from the analysis. All statistical analyses were carried out using the R statistical software package (version 2.15.1); In particular, the “nlme” package for multilevel analysis [33].

### 2.5. Research Ethics

The study was conducted as a collaborative project between the Hainan CDC and the Department of Human Ecology, University of Tokyo. The field surveys were undertaken after obtaining approval from the Ethics Committee for Medical Research at the University of Tokyo (No. 1505) and research permission from the Hainan CDC. The survey team consisted of researchers from both institutions. All participants provided informed consent prior to the survey’s administration.

## 3. Results

Table 2 shows the individual characteristics of the participants (*n* = 599). The mean age of the participants was 44.4 (SD 13.3) years. Mean BMI was 20.8 (SD 2.7) kg/m^2^. Mean urinary creatinine concentration was 1.19 (SD 0.76). The proportion of male participants was 45.1%. Those who did not graduate from junior high school comprised 33.4% of the participants, 57.4% of the participants had completed junior high school, while 9.2% had undertaken further education. The overall prevalence of smoking was 31.7%, although this behavior was overwhelmingly observed among the male participants. Just under 60% of the respondents reported using agrochemicals in the previous year. The median (interquartile range; μg/g creatinine) urinary element concentrations in all participants were: As, 73.2 (43.1–122.7); Cd, 1.8 (1.2–2.6); Pb, 3.1 (2.1–4.9); Se, 36.5 (27.2–52.3); Zn, 371 (250– 524); and Cu, 11.0 (7.9–16.1) (Table 3). 

**Table 2:** Characteristics of the participants (*n* = 599).

Variables	Mean	SD	*n*	%
**Continuous Variables**				
Age (years)	44.4	13.3		
Body Mass Index (kg/m^2^)	20.8	2.7		
Creatinine (g/L)	1.19	0.76		
**Sex**				
Male			270	45.1
**Education**				
< 9 years			200	33.4
9 years			344	57.4
> 9 years			55	9.2
**Agrochemical use in the previous month**					
Yes			356	59.4
**Current Smoking**				
Yes			190	31.7

**Table 3:** Toxic and essential trace element concentrations (μg/L).

Elements	5th Percentile	25th Percentile	Median	75th Percentile	95th Percentile
**Toxic Elements**					
As	22.5	43.1	73.2	122.7	263.1
Cd	0.71	1.20	1.79	2.60	5.24
Pb	1.29	2.12	3.09	4.93	10.14
**Trace Elements**					
Se	17.9	27.2	36.5	52.3	90.1
Zn	126	250	371	524	981
Cu	4.6	7.9	11.0	16.1	37.4

Table 4 shows the association between urinary element concentrations and individual and community characteristics among the rural residents in Hainan Island, China. Age was positively associated with As (coefficient = 0.004, *p* < 0.001), Cd (coefficient = 0.006, *p* < 0.001), Zn (coefficient = 0.003, *p* = 0.004) and Cu (coefficient = 0.002, *p* = 0.032). As, Cd and Se concentrations were higher in urine samples from females in comparison with samples from males (As: coefficient = 0.064, *p* = 0.018; Cd: coefficient = 0.107, *p* < 0.001; Se: coefficient = 0.064, *p* = 0.001), while Zn concentration was lower (coefficient = −0.062, *p* = 0.017). BMI was negatively associated with Cd concentration (coefficient = −0.013, *p* = 0.002). Greater individual involvement in the market economy was associated with higher levels of Pb (coefficient = 0.050, *p* = 0.031) and Se (coefficient = 0.054, *p* = 0.003). Community-level development was positively associated with Cd concentration (coefficient = 0.067, *p* = 0.025).

**Table 4:** The association between variation in element concentration and individual-level and community-level variables in Hainan Province, China (2008).

		As		Cd		Pb		Se		Zn		Cu	
		Coef.	*p-value*	Coef.	*p-value*	Coef.	*p-value*	Coef.	*p-value*	Coef.	*p-value*	Coef.	*p-value*
**Fixed effect (Individual level)**													
Age		0.004	< 0.001	0.006	< 0.001	0.002	0.068	< 0.001	0.554	0.003	0.004	0.002	0.032
Sex (ref = Male)	Female	0.064	0.018	0.107	< 0.001	0.007	0.769	0.064	0.001	−0.062	0.017	0.039	0.110
BMI		−0.002	0.714	−0.013	0.002	−0.005	0.239	0.003	0.299	−0.004	0.323	−0.007	0.094
Education ^a^	9 years	0.051	0.149	−0.047	0.127	−0.007	0.828	−0.025	0.285	0.030	0.379	−0.027	0.390
(ref < 9 years)	> 9 years	0.004	0.867	0.015	0.462	0.013	0.553	−0.003	0.875	0.028	0.225	−0.010	0.637
Involvement in the market economy ^b^		0.018	0.495	0.032	0.158	0.050	0.031	0.054	0.003	0.040	0.117	0.024	0.317
**Fixed effect (Community level)**													
Community-level development ^c^		0.023	0.561	0.067	0.025	0.027	0.516	0.049	0.131	−0.036	0.539	−0.001	0.985
Ethnicity (ref = Han)		0.267	0.109	0.072	0.140	−0.086	0.479	−0.102	0.212	0.030	0.854	−0.052	0.715
**Random effect**		Var.		Var.		Var.		Var.		Var.		Var.	
Individual level		0.079		0.061		0.060		0.035		0.072		0.063	
Community level		0.009		< 0.001		0.010		0.003		0.021		0.016	
Intra-class correlation (%)		10.5		0.4		14.5		7.9		22.5		20.4	

^a^ Educational attainment was categorized into three groups: <9, 9, and >9 years of schooling; ^b^ Information on respondents’ involvement in the market economy was evaluated by asking about agrochemical use in the previous year, which was dichotomized as: yes or no; ^c^ The degree of community-level economic development was based on the proportion of those who live in better housing, which resulted in the following ordering: HC (least economically developed = 1), HP, HY, HT, and HS (most economically developed = 5).

When analyzed only among the male participants, we found smoking status was positively associated with Pb concentration (coefficient = 0.090, *p* = 0.011). And the association between community-level development and Cd concentration was attenuated and no longer statistically significant (coefficient = 0.045, *p* = 0.104).

## 4. Discussion

### 4.1. Comparison of Urinary Levels with Previous Reports

Table 5 shows a comparison between the urinary toxic and trace element concentrations reported here and those reported in previous studies. 

**Table 5:** Comparison of means, medians, and selected percentiles of urinary concentrations of toxic and trace elements (μg/g creatinine) from previous studies with those from rural residents in Hainan Province, China (present study).

Element	Country	*n*	Geometric Mean	Arithmetic Mean	Median	Ref.
**As**	China (Hainan)	531	73.2	97.1	73.2	This study
	*General Populations*					
	Japan	210			114.9	[34]
	USA	13516		25.1	20.1	[35]
	UK, Healthy volunteers (Asian)	21		20.6	15.4	[36]
	UK, Healthy volunteers (Somali)	22		7.2	6.5	[36]
	UK, Healthy volunteers (White)	20		24.5	17.6	[36]
	Spain, Ria of Huelva	818	1.44	2.03	1.63	[37]
	Spain, Other Andalusian cities	816	1.26	2.05	1.5	[37]
	*Vulnerable and Other Populations*					
	USA/Mexico, Seasonal Farm Workers	258	13.2		10.7	[38]
	Mexico (Morales)	41		62.91		[5]
	Mexico (Martinez)	39		40.28		[5]
	China (Guizhou), Polluted	122	288.4			[39]
	China (Guizhou), Control	123	56.23			[39]
	China, High exposure	10	58.3			[40]
	China, Low exposure	35	23.4			[40]
	China, Exposed subjects	113		192.2		[41]
	China, Control site	30		63.6		[41]
	Taiwan, Polluted, Male	489		80.1		[42]
	Taiwan, Polluted, Female	554		88.6		[42]
	Bangladesh, Village A, Male	64	204			[23]
	Bangladesh Village A, Female	108	219			[23]
	Bangladesh Village B, Male	69	126			[23]
	Bangladesh Village B, Female	121	174			[23]
	Bangladesh, Male	4138		229.8		[43]
	Bangladesh, Female	6264		297.6		[43]
	Bangladesh, Adult	922			77	[44]
**Cd**	China (Hainan)	522	1.9	2.6	1.8	This study
	*General Populations*					
	USA	4224		0.4	0.3	[35]
	USA (non-smoker), Age: 20–39, Male		0.125			[25]
	USA (non-smoker), Age: 20–39, Female		0.179			[25]
	USA (non-smoker), Age: 40–59, Male		0.208			[25]
	USA (non-smoker), Age: 20–39, Female		0.342			[25]
	USA (non-smoker), Age: 60+, Male		0.366			[25]
	USA (non-smoker), Age: 60+, Female		0.507			[25]
	Czech Republic, Adult	657	0.29		0.31	[45]
	USA, Male	6558	0.28			[46]
	USA, Female	7398	0.4			[46]
	Spain (Ria of Huelva), Age: 18–69	818	0.49	0.65	0.57	[37]
	Spain (Other Andalusian cities)	816	0.57	0.74	0.63	[37]
	*Vulnerable and Other Populations*					
	USA/Mexico, Seasonal Farm Workers	258	0.20		0.2	[35]
	China, Exposed	75	2.62			[47]
	China, Exposed	80	1.64			[47]
	China, Non-exposed	132	0.89			[47]
	China (Guizhou), Control	123	0.86			[39]
	China (Guizhou), Polluted	122	2.16			[39]
	Belgium, Far from incinerator plant	63	0.49			[48]
	Belgium, Near incinerator plant 1	51	0.62			[48]
	Belgium, Near incinerator plant 2	33	0.43			[48]
	Spain, Incinerator plant—Far (1st)	40	0.23			[49]
	Spain, Incinerator plant—Near (1st)	46	0.37			[49]
	Spain, Incinerator plant—Far (2nd)	46	0.23			[49]
	Spain, Incinerator plant—Near (2nd)	52	0.35			[49]
	Spain, General Population	165	0.25		0.23	[50]
	Occupational Exposure	161	0.12	0.25	0.18	[51]
**Pb**	China (Hainan)	526	3.3	4.3	3.1	This study
	*General Populations*					
	USA	12092		1.6	1.2	[35]
	Spain, General Population	165	1.11		1.05	[50]
	*Vulnerable and Other Populations*					
	USA/Mexico; Seasonal Farm Workers	258	1.26		1.1	[38]
	Japan, Male (1985)	159	3.17			[52]
	Japan, Male (1993)	155	1.78			[52]
	Japan, Male (1998)	156	1.04			[52]
	Japan, Female (1985)	118	3.35			[52]
	Japan, Female (1993)	157	2.26			[52]
	Japan, Female (1998)	155	1.15			[52]
	Occupational Exposure	161	12.75	22.28	9.42	[35]
	Japan, Occupational Exposure	257		87		[26]
**Se**	China (Hainan)	529	38.3	44.7	36.5	This study
	*General Populations*					
	USA	114		83.6	76.6	[35]
	*Vulnerable and Other Populations*					
	Heroin Abuser	48		18.82		[53]
	Healthy Control	53		24.3		[53]
**Zn**	China (Hainan)	528	359	470	371	This study
	*General Populations*					
	USA	2369		371.5	297.7	[35]
	Spain (Urban + Rural)	434		698.7		[54]
**Cu**	China (Hainan)	528	11.6	15.1	11.0	This study
	*General Populations*					
	USA	4648		22.5	16.0	[35]
	Spain (Ria of Huelva)	818	8.4	11.7	8.9	[37]
	Spain (Other Andalusia cities)	816	8.6	12.6	9.0	[44]
	Spain (Urban + Rural)	434		26.6		[54]

The urinary As concentration in the present study (73.2 µg/g creatinine) fell between the range of those reported in Japan (114.9 µg/g creatinine) [34] and in the United States (20.1 µg/g creatinine) [35], the United Kingdom (6.5, 15.4 and 17.6 µg/g creatinine) [36], Spain (1.5 and 1.63 µg/g creatinine) [37] and other countries. Lindberg, *et al.* [44] reported that only 0.2% of study participants whose median urinary As concentration (77 µg/g creatinine) was equivalent to that reported in the present study had skin lesions, a typical clinical symptom of As exposure. 

Here, we should highlight that the toxicity of As largely depends on its chemical form. For example, consumers of large amounts of seafood usually display higher urinary As concentrations with few clinical symptoms because most As in seafood is organic, which is the least toxic chemical form of As [55]. Clinical studies and the identification of the chemical form of As in urine are required to determine whether the urinary As concentrations observed in Hainan Island residents indicate a need for public health measures.

Roels, *et al.* [56] concluded that individuals whose urinary Cd concentration is below 10 µg/g creatinine did not develop renal dysfunction. The urinary Cd concentrations we recorded were lower than this level. On the other hand, Cui, *et al.* [47] demonstrated that urinary indicators of renal dysfunction (*i.e.*, urinary concentrations of *N*-acetyl-β-d-glucosaminidase (NAG) and β-2-microglobulin (β_2_-MG)) were significantly higher among individuals whose urinary concentrations (2.62 and 1.64 µg/g creatinine) were equivalent to those identified in the present study. Further studies on urinary indicators of renal dysfunction are needed to ensure that the health of the local residents is sustainable.

The urinary Pb concentration was lower than levels reported in cases of occupational exposure [26,51] although a simple comparison should not be made; urinary Pb concentration reflects exposure to alkyl Pb compounds while occupational exposure to inorgnic Pb is reflected more in blood Pb concentration [57]. No investigation has determined whether clinical symptoms of Pb exposure occur at urinary Pb concentrations in the range observed in the present study.

Essential trace element uptake requirements seem to have been sufficiently satisfied among the rural residents of Hainan Island, as the trace element concentrations in urine samples fell within the ranges reported previously in developed countries. Urinary Se concentrations were lower than those reported in the United States [35], but higher than those reported in a healthy population in Tenerife, Spain [53]. Urinary Zn concentrations were lower than those reported in Spain [54], but higher than levels reported in the United States [35]. The Cu concentration in urine was equivalent to levels reported in a Spanish population [37], but lower than levels reported in the United States [35] and another Spanish population [54]. The urinary trace element concentrations reported here met nutritional requirements, and no health disorders due to trace element deficiency were likely to be present in this population.

### 4.2. Explanatory Variables for Element Concentrations

This study determined that age was positively associated with concentrations of As and Cu and marginally significantly associated with Pb. Positive associations as regards Cd and Zn were consistent with those reported in previous studies (Cd [37,38,45,50,58]; Zn [59,60]). Earlier studies have reported mixed results regarding the association between age and urinary concentrations of As [61,62], Cu [37,60] and Pb [38,60].

Sex differences were observed in urinary Zn (higher in males), As, Cd and Se (higher in females) concentrations. These findings were consistent with some of the previous studies (As [61]; Cd [45,47,50]; Se [63]; and Zn [47]) but there are also studies reporting mixed results. A negative association between BMI and Cd has been reported previously [46] and was also detected in the present study.

None of the agrochemical products which the participants stated that they used (such as butachlor, glyphosate and paraquat) contained toxic heavy elements (As, Cd, or Pb). This suggests the association observed between agrochemical usage, which we categorized as a marker of individual involvement in the market economy, and urinary Pb concentrations must have resulted from unknown behavioral factors associated with economic development. The elevated urinary Se concentrations observed among those with a higher degree of involvement in the market economy might have been the result of the adoption of new dietary habits (e.g., greater meat consumption, which might have increased with agrochemical usage and cash income). 

While smoking is considered to be an important source of toxic elements (Cd and Pb), especially in China [64], this study revealed that smoking itself was only significantly associated with urinary Pb concentrations in male participants. Although the association between the degree of community-level economic development and Cd concentration was attenuated when we incorporated smoking status into the statistical model, the community-level variation in Cd concentration might not have been explained solely by the proportion of smokers in these communities, but rather, it might have also been due to other factors (e.g. the use of batteries); however, we do not have data to test this supposition.

The earlier finding that cigarette smoking was negatively associated with Se [65] was observed in this study but at borderline significance (B = −0.05, *p* = 0.066), possibly due to the relatively low statistical power.

### 4.3. Limitations

This research has several limitations. First, the cross-sectional study design prevented the determination of causal relationships. Changes in exposure might not only be due to the economic changes we hypothesized based on our observations during the survey. Second, elemental concentrations in the natural environment or ordinary diet were not evaluated. Because 60% of As on the ground surface originates from natural sources [7], variations in the element’s natural background levels might have influenced the results. Third, although the authors selected communities with different degrees of economic development following discussions with the local authorities, those communities might not have been representative of all the communities on Hainan Island. Fourth, urinary Zn, Pb, and Cu levels are not common biomarkers because their sensitivity is not sufficient for clinical purposes. The urinary Zn level has a limited capacity to predict Zn depletion in the body compared with 24-h urine collection [66]. Some previous reports have questioned the use of urine samples to evaluate individual exposures to Pb and Cu [67,68]. However, their use is justified because urinary concentrations are known to react to changes in exposure/intake levels at the population level [26].

## 5. Conclusions

The degree of economic development at the community-level was positively associated with urinary cadmium concentration, while greater individual involvement in the market economy was positively associated with lead and selenium. Although the urinary concentration of toxic metals we recorded in this study was unlikely to cause health disorders, close attention should be paid to these measures in the future to help ensure that the health of local residents who experience rapid economic development is sustainable. 
